# Diagnostic performance and interchangeability of two commercial anti-RSV−IgG and IgA ELISAs across clinically relevant populations

**DOI:** 10.1128/spectrum.00417-26

**Published:** 2026-06-26

**Authors:** Rainer Gosert, Svenia Schmid, Laura Infanti, Andreas Holbro, Andreas Buser, Jakob Passweg, Jörg Halter, Jonas Lötscher, Carla S. Walti, Sarah Tschudin-Sutter, Nina Khanna, Ulrich Heininger, Karoline Leuzinger

**Affiliations:** 1 Clinical Virology, University Hospital Basel 30262 , Basel, Switzerland; 2 Regional Blood Transfusion Service, Swiss Red Cross, Basel, Switzerland; 3 Division of Hematology, University Hospital Basel 30262 , Basel, Switzerland; 4 Department of Biomedicine, University of Basel 27209, Basel, Switzerland; 5 Division of Infectious Diseases, University Hospital Basel 30262 , Basel, Switzerland; 6 Department of Clinical Research, University Hospital Basel and University of Basel 27209, Basel, Switzerland; 7 Department of Paediatric Infectious Diseases and Vaccinology, University of Basel Children’s Hospital, Basel, Switzerland; Naturwissenschaftliches und Medizinisches Institut an der Universitat Tubingen, Reutlingen, Germany

**Keywords:** ELISA, anti-RSV IgA, anti-RSV IgG, prefusion F antibodies, serological surveillance, respiratory syncytial virus, RSV

## Abstract

Accurate assessment of anti-respiratory syncytial virus (RSV)-specific antibodies has gained importance following the introduction of RSV vaccines and long-acting monoclonal antibody prophylaxis. However, the analytical performance and interchangeability of commercially available RSV−ELISAs remain insufficiently defined, leaving uncertainty about their reliability for clinical diagnostic practice. We performed a head-to-head comparison of the widely used commercial Euroimmun− and Virotech‒ELISA for detection of total anti-RSV‒IgG and IgA in 462 sera from four cohorts: healthy adults (*n* = 100), pregnant women (*n* = 59), children (*n* = 101), and vaccinated transplant recipients (*n* = 101; baseline and day + 30). Prefusion-F (pre-F)-specific IgG was quantified to infer functionally relevant antibody responses. Qualitative agreement, quantitative bias, correlations, and age-dependent patterns were assessed. Qualitative concordance in anti-RSV‒IgG detection was high (83.8%) between assays. However, the Virotech‒ELISA systematically yielded higher IgG detection rates and quantitative values (*P* < 0.001). Total anti-RSV‒IgG correlated significantly with pre-F‒IgG in all cohorts, with strongest correlations in children (r_s_ = 0.92) and vaccinated transplant recipients (r_s_ = 0.79). Anti-RSV‒IgA was detected infrequently (6%–11%), with moderate (79.4%) inter-assay concordance. IgG‒IgA correlations were weak in healthy adults and pregnant women (r_s_ < 0.25), but moderate in children and vaccinated transplant recipients (r_s_ ≥ 0.40). The Euroimmun− and Virotech‒ELISAs are not analytically interchangeable and exhibit systematic assay-dependent differences. Integrated analysis of total anti-RSV‒IgG, pre-F‒IgG, and IgA across clinically relevant populations emphasizes the need for assay- and population-aware interpretation to support reliable use of RSV serology in seroepidemiological studies and diagnostic laboratory practice.

**IMPORTANCE:**

With the recent introduction of espiratory syncytial virus (RSV) vaccines and infant monoclonal antibody prophylaxis, serological testing is gaining relevance in vaccine immunogenicity trials, post-licensure evaluations, and population-based serosurveillance. Although commercial ELISAs offer the potential to harmonize antibody testing, their appropriate application requires a detailed understanding of assay-specific performance and calibration characteristics. Inadequate validation can limit inter-laboratory comparability, such that identical samples yield different quantitative results across centers, thereby complicating longitudinal antibody monitoring and harmonized analysis in multicenter surveillance and vaccine studies. In this context, the present study provides a systematic cross-comparison of two widely used RSV−ELISAs and demonstrates that, despite good qualitative agreement, they are not quantitatively interchangeable and exhibit systematic differences in IgG and IgA measurements across populations and immune contexts. By characterizing the performance of total IgG, pre-F−specific IgG, and IgA in diverse clinical settings, this work supports informed assay selection and interpretation of RSV serology in clinical diagnostic practice.

## INTRODUCTION

Respiratory syncytial virus (RSV) is a major cause of respiratory tract infection in young children, older adults, and immunocompromised individuals, contributing to millions of hospitalizations and substantial global mortality each year (1–5). RSV expresses the fusion (F) protein as a major surface glycoprotein and the primary target of virus-neutralizing antibodies, forming the antigenic basis of current vaccine and monoclonal antibody strategies (6–8). During the study period, two pre-F−based RSV vaccines, Arexvy (GSK) and Abrysvo (Pfizer), were licensed in Switzerland for older adults, and Abrysvo was additionally approved for maternal immunization (9–12). In parallel, the long-acting monoclonal antibody nirsevimab (Sanofi/AstraZeneca) has been recommended for universal infant prophylaxis (13–15). Although RSV-neutralizing and serologic responses after nirsevimab administration have been characterized in infants, baseline serologic data from nirsevimab-naïve pediatric cohorts remain important for contextualizing post-implementation changes (15, 16). With the rapid implementation of RSV prevention strategies, robust serological tools are essential to define baseline immunity, evaluate vaccine-induced antibody responses, while post-licensure monitoring should be integrated with molecular surveillance of circulating RSV strains to detect emerging sequence variation that may contribute to vaccine-relevant escape (17). Beyond surveillance, serological assessment allows quantification of vaccine-induced neutralizing antibody responses, comparison of immune responses across patient populations, and supports efforts to define protective antibody thresholds (18–21). In this setting, whole-virus antigen assays remain relevant in RSV serology because they capture polyclonal antibody responses to multiple viral antigens and thus provide a pragmatic measure of cumulative prior RSV exposure. They are also established in diagnostic and seroepidemiologic workflows, allowing comparison with existing assay frameworks and historical data sets. In contrast, pre-F-specific IgG more directly reflects neutralizing and vaccine-relevant immunity. These readouts are therefore complementary rather than interchangeable. Serum IgA primarily reflects recent antigenic stimulation or immune boosting and should not be interpreted as a stand-alone marker of acute RSV infection, for which nucleic acid testing remains preferred.

Despite the expanding use of RSV serology in vaccine studies, clinical evaluation, and population-based surveillance, the analytical performance and quantitative comparability of commercially available ELISA assays remain insufficiently defined. Platform-specific differences in antigen composition, calibration strategies, and signal interpretation may influence antibody measurements and limit comparability between diagnostic laboratories. Consequently, identical patient samples may yield different quantitative results across diagnostic centers, complicating longitudinal assessment of antibody kinetics and harmonized analysis in multicenter studies.

To address this gap, the present study performed a direct head-to-head comparison of two widely used commercial ELISAs for detecting and quantifying anti-RSV−IgG and IgA across four cohorts, healthy blood donors, pregnant women, children, and allogeneic hematopoietic cell transplant (HCT) recipients following RSV vaccination. The study evaluated qualitative agreement, quantitative bias, and cohort-dependent correlations between assays. In addition, pre-F-specific IgG was quantified in a subset of sera to contextualize total IgG measurements with functionally relevant antibody responses across distinct immune settings.

## MATERIALS AND METHODS

### Study design and patient cohorts

This single-center retrospective study was conducted at the University Hospital Basel, a tertiary care center in northwestern Switzerland. Serum samples from four predefined cohorts were analyzed using two commercial anti-RSV−IgG and IgA ELISAs, and an anti-RSV pre-F-IgG immunoassay. The study cohorts represented a convenience sample derived from routine clinical and diagnostic workflows. Cohort 1 comprised 100 healthy adult blood donors sampled between November and December 2023. Cohort 2 consisted of 59 pregnant women in the first trimester who were sampled between October and December 2023. Neither cohort had received RSV vaccination at the time of sampling. Cohort 3 included 101 children whose serum samples were collected between June 2023 and February 2024. None had received nirsevimab at the time of sampling. Cohort 4 included 101 allogeneic HCT-recipients whose samples were obtained at baseline (day 0) and at day 30 after administration of the pre-F-based vaccine Arexvy between December 2023 and December 2024 (19). Table S1 summarizes the RSV vaccines and monoclonal antibodies approved or available in Switzerland during cohort enrollment.

### RSV-specific immunoassays

All serum samples were stored at −20°C until analysis. Anti-RSV−IgG and IgA were quantified using the commercial Euroimmun anti-RSV−IgG and IgA ELISAs (EUROIMMUN AG, Lübeck, Germany) and the Virotech anti-RSV−IgG and IgA ELISAs (Virotech Diagnostics GmbH, Rüsselsheim, Germany) on the DS2 system (Dynex Technologies, VA, USA). A predefined subset of sera from each cohort underwent anti-RSV pre-F-specific IgG testing (ACROBiosystems, Newark, DE, USA) on the Epoch spectrophotometer (BioTek Instruments, VT, USA) to enable comparison between total anti-RSV−IgG and pre-F-specific immune responses. All immunoassays were performed at the Clinical Virology unit, University Hospital Basel, according to the manufacturers’ instructions, and results were interpreted using the assay-specific cutoffs (Table S2).

### Statistical analysis

Statistical analyses and visualizations were performed in GraphPad Prism (version 10; GraphPad Software, CA, USA). RSV antibody readouts, including total anti-RSV−IgG, pre-F−IgG, and IgA, were assessed using Wilcoxon matched-pairs test and Spearman rank correlation. Agreement between quantitative measurements across immunoassays was evaluated by Bland−Altman analyses.

## RESULTS

A total of 462 sera from 361 individuals across four predefined study cohorts were included in the analysis, including two time points for cohort 4 (Table 1). Anti-RSV−IgG and IgA were measured in all sera using two commercial ELISA platforms, and anti-RSV pre-F-specific IgG was quantified in a subset of 327 sera.

**TABLE 1:** Demographic characteristics of the study cohorts^*a*^

Characteristic	Cohort 1 (healthy adults)	Cohort 2 (pregnant women)	Cohort 3 (children)	Cohort 4 (HCT recipients)
Patients (*n*)	100	59	101	101
Serum samples (*n*)	100	59	101	202
Female (*n*, %)	53 (53.0%)	59 (100%)	41 (40.6%)	37 (36.6%)
Age in years (median, Q_1_ and Q_3_)	52 (37, 61)	35 (31, 37)	9 (5, 13)	64 (57, 70)
Age in years (min and max range)	20–75	16–45	1–17	21–78

^*a*^ HCT, hematopoietic cell transplant; Q_1_, 25^th^ quartile; Q_3_, 75^th^ quartile.

### Comparison of anti-RSV−IgG detection by two ELISAs across study cohorts

**Fig 1:**
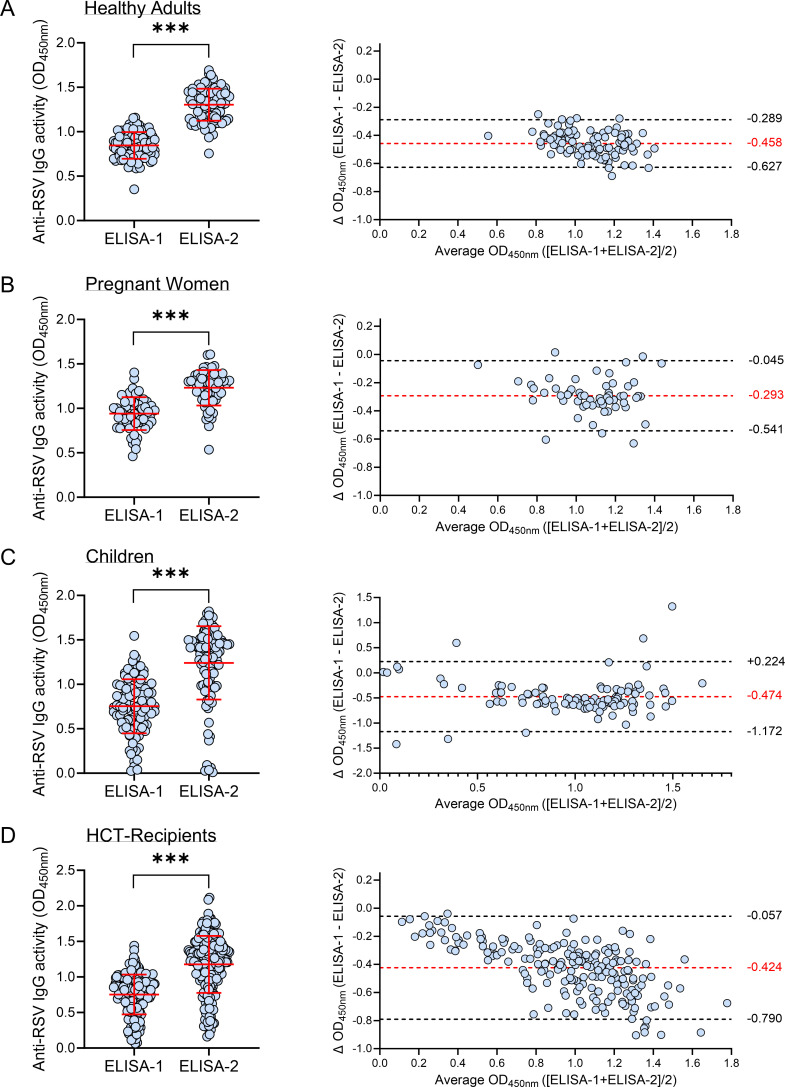
Comparison of anti-RSV−IgG antibody activity by two commercial ELISAs Anti-RSV−IgG optical density (OD_450nm_) values were measured using the Euroimmun anti-RSV−IgG ELISA (ELISA-1) and the Virotech anti-RSV−IgG ELISA (ELISA-2) in 462 sera from 361 individuals across four predefined study cohorts. Median optical density values with interquartile ranges are shown, and paired comparisons were performed using the Wilcoxon matched-pairs test (left panels). Bland−Altman analyses are shown, with the dashed red line indicating the mean bias and the dashed black lines indicating ±1.96 standard deviations (right panel). (**A**) Sera from 100 healthy adult blood donors (cohort 1). (**B**) Sera from 59 pregnant women (cohort 2). (**C**) Sera from 101 children (cohort 3). (**D** ) Sera from 101 allogeneic hematopoietic cell transplant recipients sampled at baseline (day 0) and 30 days post-vaccination (day 30), yielding 202 samples (cohort 4). Statistical significance was assessed using the Wilcoxon matched-pairs test; ***P < 0.001.

Across the four cohorts, qualitative anti-RSV−IgG classification showed substantial agreement between Euroimmun−ELISA and Virotech−ELISA, with an overall concordance of 83.8%. Concordance was highest in healthy adults (92.0%) and children (87.1%), followed by allogeneic HCT-recipients (84.2%), and was lowest in pregnant women (62.7%). In all cohorts, Virotech−ELISA classified more sera as anti-RSV−IgG-positive than Euroimmun−ELISA, predominantly among partially discordant samples classified as indeterminate by Euroimmun−ELISA (Fig. S1). In quantitative analyses, Virotech−ELISA consistently yielded higher anti-RSV−IgG than Euroimmun−ELISA in all four cohorts (*P* < 0.0001; Fig. 1). Bland−Altman analyses demonstrated a systematic negative bias of Euroimmun−ELISA, with mean differences ranging from −0.293 to −0.474 OD units (Fig. 1). In allogeneic HCT recipients, this pattern remained consistent at both baseline (day 0) and day 30 post-vaccination, with higher anti-RSV−IgG values measured by Virotech−ELISA at both time points (Fig. S2A and B).

### Comparison of anti-RSV−IgA detection by two ELISAs across study cohorts

**Fig 2:**
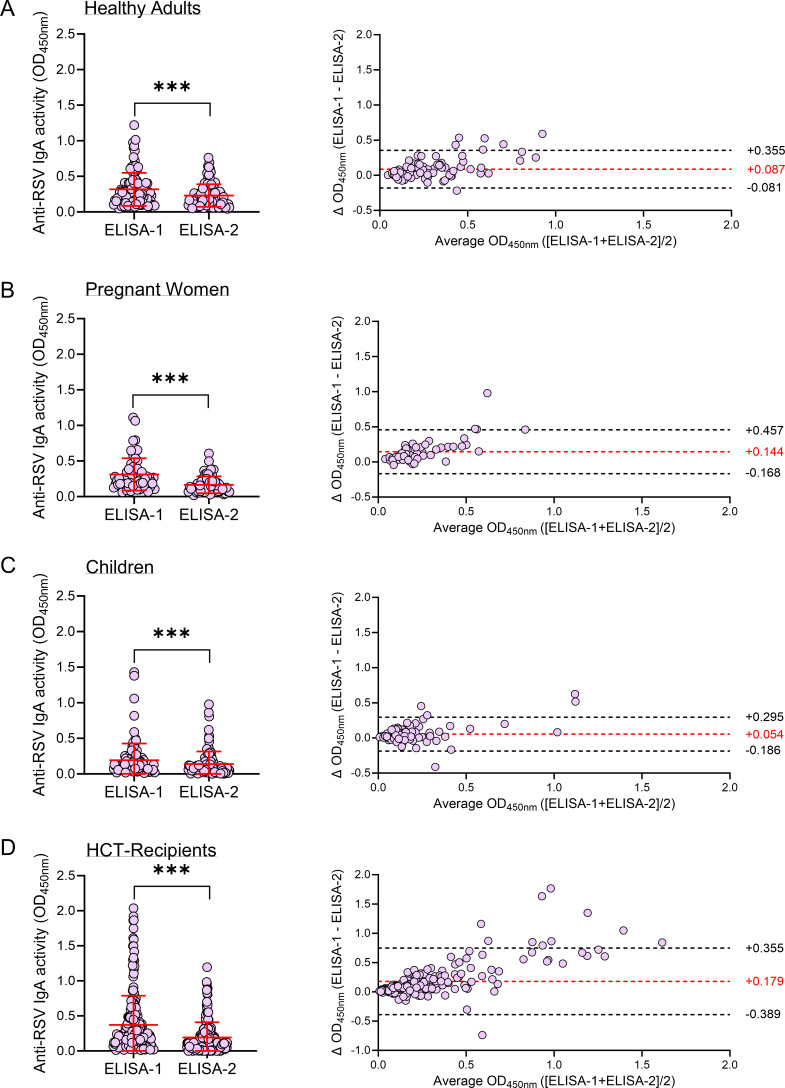
Comparison of anti-RSV−IgA antibody activity by two commercial ELISAs. Anti-RSV−IgA optical density (OD_450nm_) values were measured using the Euroimmun anti-RSV−IgA ELISA (ELISA-1) and the Virotech anti-RSV−IgA ELISA (ELISA-2) in 462 sera from 361 individuals across four predefined study cohorts. Median optical density values with interquartile ranges are shown, and paired comparisons were performed using the Wilcoxon matched-pairs test (left panels). Bland−Altman analyses are shown, with the dashed red line indicating the mean bias and the dashed black lines indicating ±1.96 standard deviations (right panel). (**A**) Sera from 100 healthy adult blood donors (cohort 1). (**B**) Sera from 59 pregnant women (cohort 2). (**C**) Sera from 101 children (cohort 3). (**D**) Sera from 101 allogeneic hematopoietic cell transplant recipients sampled at baseline (day 0) and 30 days post-vaccination (day 30), yielding 202 samples (cohort 4). Statistical significance was assessed using the Wilcoxon matched-pairs test; ***P < 0.001.

Qualitative anti-RSV−IgA classification showed moderate agreement between Euroimmun−ELISA and Virotech−ELISA, with an overall concordance of 79.4%. Concordance was comparable across cohorts, with agreement rates of 79.0% in healthy adults, 78.0% in pregnant women, 87.1% in children, and 76.2% in allogeneic HCT recipients. Most sera were classified as anti-RSV−IgA-negative by both assays. Euroimmun−ELISA classified a higher number of sera as anti-RSV−IgA-positive or indeterminate than Virotech−ELISA, particularly in healthy adults and children (Fig. S3). Quantitative comparison showed that Euroimmun−ELISA yielded higher anti-RSV−IgA values across all cohorts (*P* < 0.0001; Fig. 2). Bland−Altman analyses demonstrated a consistent positive bias for Euroimmun−ELISA, with mean differences ranging from 0.054 to 0.179 OD units (Fig. 2). In allogeneic HCT recipients, this relationship was likewise maintained at baseline (day 0) and day 30 post-vaccination, with higher anti-RSV−IgA values measured by Euroimmun−ELISA at both time points (Fig. S2C and D).

### Correlation between total anti-RSV−IgG and pre-F−IgG

**Fig 3:**
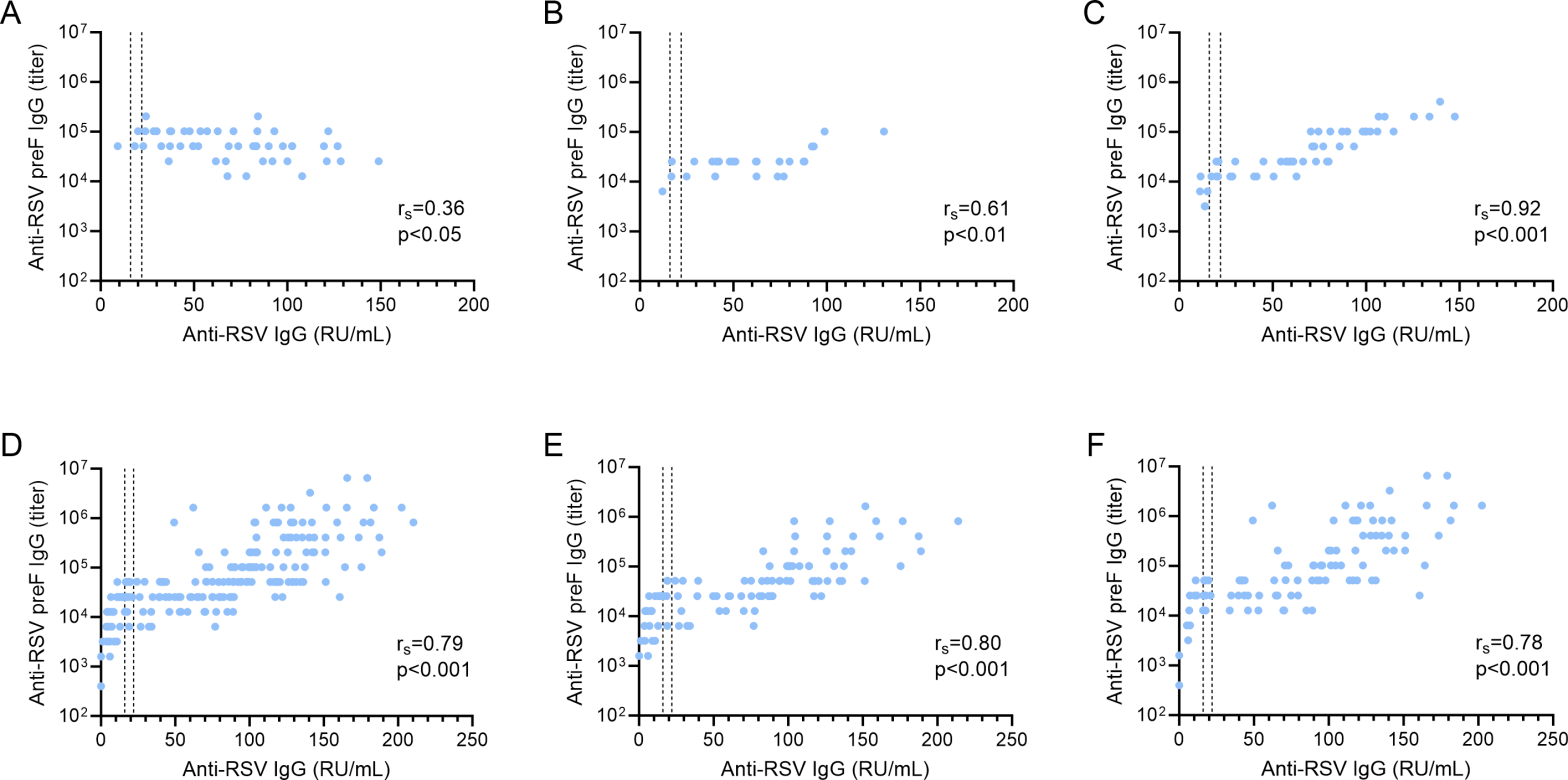
Correlation between total anti-RSV−IgG and prefusion F-specific IgG titers quantitative comparison of total anti-RSV−IgG measured by the euroimmun−ELISA and prefusion F-specific IgG titers in 327 sera from four study cohorts. Dotted lines indicate assay-specific cutoff values for indeterminate and positive results. (**A**) Sera from 50 healthy adult blood donors (cohort 1). (**B**) Sera from 25 pregnant women (cohort 2). (**C**) Sera from 50 children (cohort 3). (**D**) All 202 serum samples from allogeneic hematopoietic cell transplant recipients, including baseline and post-vaccination samples (cohort 4). (**E**) Sera from 101 allogeneic hematopoietic cell transplant recipients at baseline (day 0). (**F**) Sera from 101 allogeneic hematopoietic cell transplant recipients at 30 days post-vaccination (day 30).

Pre-F−IgG was quantified in a subset of sera as a proxy for RSV neutralizing activity (7, 22–24) and correlated with total anti-RSV−IgG quantified by the Euroimmun−ELISA across cohorts (Fig. 3). Significant correlations were observed in all groups, with moderate associations in healthy adults (r_s_ = 0.36, *P* < 0.05) and pregnant women (r_s_ = 0.61, *P* < 0.01), and stronger correlations in children (r_s_ = 0.92, *P* < 0.001) and allogeneic HCT recipients (r_s_ = 0.79, *P* < 0.001). Median pre-F−IgG titers differed across cohorts. In healthy adults, median titers were 51,200 (IQR, 44,800–102,400), whereas pregnant women showed lower median titers of 25,600 (IQR, 12,800–25,600). Children also exhibited a median titer of 25,600, with a wider distribution (IQR, 12,800–102,400), indicating more heterogeneous infection histories. In allogeneic HCT-recipients, median pre-F−IgG titers were higher overall at 51,200 (IQR, 25,600–204,800). Longitudinal analysis in this cohort demonstrated a significant increase following vaccination, with median titers rising from 25,600 at baseline (IQR, 19,200–102,400) to 51,200 at day 30 (IQR, 25,600–409,600). Correlations between total anti-RSV−IgG and pre-F−IgG remained strong at both timepoints, with rₛ values of 0.80 at baseline and 0.78 at day 30 (both *P* < 0.001; Fig. 3E and F).

Pre-F-specific IgG titers were evaluated in 50 children aged 1 to 17 years (Fig. S4). No significant correlation was observed between age and pre-F−IgG titers (r_s_ = 0.23, *P* = 0.11), reflecting a broad overall distribution rather than a linear age-dependent trend. In children aged 1 to 5 years, pre-F−IgG titers spanned a wide range (range, 6,400–409,600), indicating marked interindividual heterogeneity. Two-thirds of children in this age group exhibited titers ≥25,600, potentially associated with recent RSV exposure. Between 6 and 10 years of age, titers remained broadly distributed (range, 3,200–102,400), suggesting ongoing variability related to the timing and frequency of recurrent RSV reinfections. In contrast, from 11 to 15 years of age, interindividual variability decreased, with titers converging and clustering predominantly between 12,800 and 102,400. Among adolescents aged 16 to 17 years, pre-F−IgG titers remained within a similar range (range, 12,800–204,800), with minimal further variability. This convergence of titers is consistent with plateauing of pre-F−IgG concentrations following cumulative RSV exposure and indicates relative stabilization of antibody levels beyond early adolescence.

### Correlation between anti-RSV−IgG and IgA levels

Correlations between total anti-RSV−IgG and IgA concentrations varied by cohort and ELISA assay. For the Euroimmun−ELISA, correlations were low and not statistically significant in healthy adults (r_s_ = 0.12, *P* = 0.25), pregnant women (r_s_ = 0.08, *P* = 0.56), and children (r_s_ = 0.16, *P* = 0.11), whereas a modest but significant correlation was observed in allogeneic HCT-recipients (r_s_ = 0.34, *P* < 0.001; Fig. S5). For the Virotech−ELISA, correlations were similarly low in healthy adults (r_s_ = 0.11, *P* = 0.26) and pregnant women (r_s_ = 0.24, *P* = 0.07) but were significant in children (r_s_ = 0.40, *P* < 0.001) and strongest in allogeneic HCT recipients (r_s_ = 0.56, *P* < 0.001; Fig. S6). In longitudinal analyses of vaccinated allogeneic HCT recipients, IgG−IgA quantitative correlation increased following vaccination, with correlation coefficients rising from r_s_ = 0.52 at baseline (day 0) to r_s_ = 0.58 at day 30 (*P* < 0.001; Fig. S6E and F).

### Age-stratified anti-RSV−IgG and IgA seroprevalence in children

**Fig 4:**
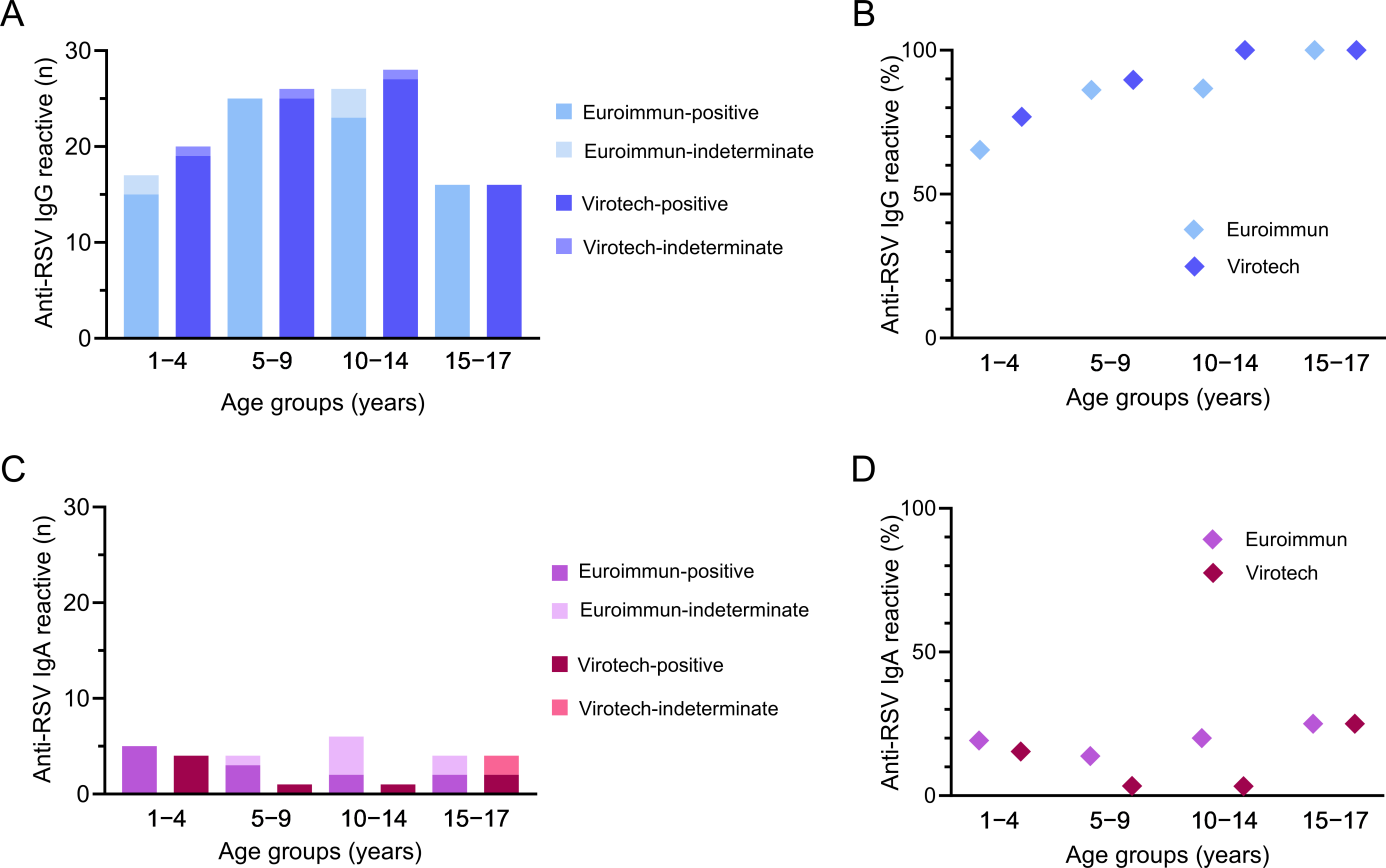
Age-stratified anti-RSV−IgG and IgA reactivity in children Anti-RSV−IgG and IgA were assessed in 101 pediatric serum samples (cohort 3) using Euroimmun− and Virotech‒ELISAs. Age groups were 1–4 years (*n* = 26), 5–9 years (*n* = 29), 10–14 years (*n* = 30), and 15–17 years (*n* = 16). Number of sera classified as positive (dark shading) or indeterminate (light shading) for anti-RSV−IgG (**A**) and anti-RSV−IgA (**C**), stratified by age group. The percentage of sera classified as positive or indeterminate for anti-RSV−IgG (**B**) and anti-RSV−IgA (**D**), stratified by age group.

Among children, age-stratified qualitative anti-RSV−IgG and IgA results showed broadly similar patterns across the Euroimmun− and Virotech−ELISAs, although discordant classifications were mainly observed in reactive borderline or indeterminate samples, particularly in younger age groups (Fig. 4A through C). Anti-RSV−IgG seroprevalence increased with age, from 76.9% in children aged 1 to 4 years to 89.7% in those aged 5 to 9 years and reached 100% in children aged ≥10 years (Fig. 4B). This pattern is consistent with cumulative RSV exposure during early childhood and near-universal seroconversion by adolescence. In contrast, anti-RSV−IgA seroprevalence remained low across all pediatric age groups and showed no clear age-dependent increase, with modest assay-dependent variation across age strata. Anti-RSV−IgA positivity was detected in 19.2% of children aged 1 to 4 years, 13.8% in those aged 5 to 9 years, ≥20.0% in children aged ≥10 years (Fig. 4D). These relatively stable and low IgA seroprevalence rates across age strata are consistent with the transient nature of RSV-specific IgA responses and their dependence on recent or ongoing exposure rather than cumulative immunity.

## DISCUSSION

This study provides a dedicated head-to-head comparison of two widely used commercial RSV ELISAs for anti-RSV−IgG and IgA detection. Pre-F-specific IgG was quantified in a subset of participants to contextualize total antibody measurements in relation to functionally relevant neutralizing immunity. Across all cohorts, the Euroimmun− and Virotech‒ELISAs showed high qualitative concordance (83.8%) for anti-RSV−IgG detection, indicating overall agreement in serostatus classification. However, systematic quantitative differences indicated that the immunoassays are not interchangeable for quantitative IgG assessment. The Virotech‒ELISA consistently yielded higher anti-RSV−IgG values and more frequently classified low-titer or borderline samples as positive, resulting in assay-dependent serostatus assignment. Although both assays use RSV-A Long strain whole-virus lysate derived from Vero cells, assay-specific differences in antigen preparation, plate coating, calibrator design, and signal normalization may contribute to the observed quantitative divergence (Table S2). More generally, prior studies have shown that the host cell system used for RSV propagation can alter glycosylation of the attachment G protein (25, 26), and thereby affect epitope exposure and antibody binding (26–28), underscoring the methodological sensitivity of whole-virus serology to antigen production conditions. Differences in calibration further contribute to quantitative divergence, as Euroimmun reports relative units per milliliter using internal calibrators, whereas Virotech expresses results relative to a kit-specific cutoff control (Table S2). The WHO international standard for antiserum to RSV was not used in the present study (29). Accordingly, absolute anti-RSV−IgG values remain only partly harmonized across platforms because both assays relied on manufacturer-specific calibrators. This limitation mainly affects cross-study and cross-laboratory comparison rather than the internal assay comparison performed here. Future incorporation of the WHO standard may further improve inter-assay harmonization and strengthen the comparability of quantitative anti-RSV−IgG measurements in diagnostic laboratory practice.

Despite these analytical differences, total anti-RSV−IgG quantified by the Euroimmun−ELISA correlated significantly with pre-F−IgG titers across cohorts, with clear population-dependent effects. Strong correlations were observed in children and in allogeneic HCT recipients following vaccination with the adjuvanted pre-F−based vaccine Arexvy (19). In these settings, recent antigenic exposure through primary infection, reinfection, or vaccination likely increases the contribution of functionally relevant anti-F responses to the total anti-RSV−IgG signal, thereby strengthening the observed relationship between total anti-RSV−IgG and pre-F−IgG (6, 19). This interpretation is supported by studies in infants with acute RSV infection showing that pre-F antibodies predominate over those targeting postfusion-F and exhibit the greatest neutralizing activity (6). Consistently, higher neutralizing antibody levels have been associated with protection against RSV disease in observational studies and confirmed in clinical trials of pre-F-based vaccines and monoclonal antibody prophylaxis (9–15). In contrast, correlations were weaker in healthy adults and pregnant women, likely reflecting heterogeneous antibody repertoires shaped by remote exposures, immune maturation, and antibody waning over time (30). Longitudinal studies show that pre-F−IgG declines markedly within six months following RSV infection (24), reducing the dynamic range of pre-F antibodies in the absence of recent boosting. In addition, cumulative lifelong RSV exposures generate complex polyclonal antibody repertoires, in which historical responses to postfusion-F and attachment G-proteins may contribute disproportionately to total IgG, further weakening correlations with pre-F−directed immunity. This interpretation aligns with mechanistic observations that postfusion-F, and shared-epitope antibodies account for a substantial fraction of adult binding antibodies yet contribute little to functional neutralization (7).

Pregnancy introduces additional physiological and immunological changes that can further decouple total IgG levels from protective pre-F−specific immune responses. Hemodilution, placental IgG transfer, hormonal modulation, and immunological modulation can all influence circulating IgG levels independent of antigen specificity (31, 32). Accordingly, total anti-RSV−IgG during pregnancy may not reliably reflect the magnitude of functionally protective immunity. Supporting this distinction, previous studies have shown that only pre-F-specific antibody titers are associated with infant disease severity, with significantly lower pre-F−IgG titers observed in mothers of infants hospitalized with RSV bronchiolitis (33). These considerations are also relevant to passive protection of the newborn, which depends not only on maternal antibody specificity but also on the timing and persistence of maternal antibody levels during pregnancy. Recent evidence suggests that maternal RSV-specific IgG levels during pregnancy may be influenced by gestational timing and antibody kinetics. Notably, a recent study reported a decline in anti-RSV−IgG concentrations from antenatal to delivery samples, indicating that maternal antibody levels may wane prior to birth (34). Lower maternal antibody levels near term could reduce the amount of RSV-specific IgG available for transplacental transfer and thereby influence the degree of passive protection conferred to the newborn. These findings underscore the importance of optimizing vaccination timing during pregnancy to maintain robust maternal RSV-specific antibody levels near term.

Whereas pre-F−IgG reflects the presence of functionally relevant antibody populations that may persist after natural infection or vaccination, anti-RSV−IgA responses are transient and closely linked to recent antigenic stimulation (35, 36). Serum IgA should therefore be interpreted primarily as a marker of recent exposure or immune boosting, rather than as a stand-alone diagnostic marker of acute RSV infection. This interpretation is consistent with controlled human infection studies showing that pre-existing nasal IgA, rather than serum IgG, represents the strongest antibody correlate of protection against RSV infection, with higher mucosal IgA levels associated with reduced infection risk, lower viral loads, and milder disease courses (36–39). Importantly, natural RSV infection induces limited and inconsistently detectable IgA-secreting memory B-cell responses, particularly in infants and children, whereas IgG memory responses are more robust and durable (30, 36). Consequently, anti-RSV−IgA wanes rapidly even when serum IgG levels remain stable (35, 36). This biology explains the low IgA prevalence and weak or absent IgG−IgA correlations observed in healthy adults and pregnant women and indicates that absence of detectable serum IgA reflects lack of recent RSV exposure rather than immunological naïveté (36, 40). In contrast, children and recently vaccinated allogeneic HCT recipients showed higher IgA detection rates and stronger IgG−IgA correlations, consistent with recent or ongoing antigenic stimulation. In young children, primary infections and early reinfections lead to transient serum IgA that reflects recent RSV exposure (41). Because maternal IgA is not transferred across the placenta, serum IgA in infants and young children represents endogenous immune responses. Population-based studies indicate that approximately 40% of 1-year-old children remain IgA seronegative, whereas by 3 years of age, nearly all show serological evidence of prior RSV exposure (42). Within this cohort, age-dependent patterns of RSV humoral immunity reflected both recent exposure and cumulative immune maturation. During early childhood, serum IgA was detected infrequently, while pre-F−IgG levels remained broadly variable up to approximately 10 years of age, reflecting heterogeneous timing of primary infection and early reinfections. By approximately 10 to 15 years of age, pre-F−IgG titers converged toward a stable plateau, coinciding with high overall IgG seroprevalence after cumulative RSV exposure. These patterns align with population-based serosurveillance data demonstrating rapid decay of maternal IgG in infancy, initiation of endogenous IgG production from 3 to 6 months of age, and near-universal RSV infection by early childhood (42).

Taken together, these findings demonstrate that RSV antibody specificities capture distinct aspects of exposure and immune history. Total anti-RSV−IgG measured by whole-virus ELISAs primarily reflects cumulative exposure, whereas pre-F-specific IgG more directly represents functionally relevant and potentially neutralizing immunity. After recent antigenic exposure, pre-F-directed antibodies constitute a substantial fraction of the anti-RSV−IgG response, allowing total IgG to approximate protective immunity. In unboosted adults, heterogeneous immune histories and antibody waning attenuate this relationship, necessitating cautious interpretation in the context of the patient population. Serum IgA represents a transient marker of recent infection or immune boosting and is therefore most informative in pediatric populations and recently immunized individuals. When applied in an assay- and population-specific manner, combined assessment of total IgG, pre-F−IgG, and IgA can provide a nuanced characterization of RSV immune status, including recent exposure, cumulative immunity, and functionally relevant antibody profiles. At the same time, because pre-F is a highly relevant vaccine antigen, pre-F-based IgG serology alone may not fully distinguish vaccine-induced responses from those arising after natural infection. Future studies may therefore benefit from broader antigen panels, including RSV G- and N-based readouts, ideally within multiplex or other high-throughput platforms such as electrochemiluminescence immunoassays, to improve discrimination between vaccination and prior infection and to broaden serologic interpretation (43–45).

### Limitations

This study has some limitations. First, it was conducted at a single center in Switzerland and included cohorts sampled across different calendar periods between 2023 and 2024, which may limit generalizability to populations with different ethnic, geographic, or exposure backgrounds. Although all samples were collected after the SARS-CoV-2 pandemic, when RSV circulation had largely returned to pre-pandemic patterns (46), differences in exposure timing between cohorts cannot be excluded. This limitation is particularly relevant for serum IgA, which reflects recent antigenic stimulation and is sensitive to variation in sampling intervals. Second, detailed clinical and virological data were unavailable. Information on the number and timing of prior RSV infections, RSV nucleic acid testing, and clinical symptoms was not collected, precluding assessment of the interval between RSV infection and sampling. Third, immunological analyses were limited in scope. Neutralizing activity was inferred from pre-F−IgG rather than directly measured using live virus or pseudovirus neutralization assays. Serum IgA was assessed without paired mucosal samples, preventing direct evaluation of mucosal immunity. Finally, the analysis focused on humoral immune responses and did not assess antibody effector functions or cellular immune responses.

### Conclusion

This study provides a systematic head-to-head evaluation of two widely used commercial RSV ELISAs and demonstrates that these assays are not analytically interchangeable.

Although qualitative agreement for anti-RSV−IgG detection was high, systematic assay-dependent differences in quantitative readouts were observed, reflecting differences in antigen composition, calibration strategies, and signal interpretation. These findings underscore that RSV serological results require assay-specific interpretation and that direct comparison of quantitative values across platforms is inappropriate in the absence of harmonized reference standards. By systematically characterizing assay performance across multiple clinically relevant populations, this work provides an evidence-based framework to guide selection and interpretation of RSV ELISAs in clinical diagnostic practice, seroepidemiological surveillance, and longitudinal monitoring of vaccine-induced immunity.

## Data Availability

The data supporting the findings of this study are available from the corresponding author upon reasonable request.

## SUPPLEMENTAL MATERIAL

Figure S1 spectrum.00417-26-s0001.pdf Flow chart illustrating concordance in anti-RSV IgG detection by Euroimmun‒ and Virotech‒ELISAs across four study cohorts: healthy blood donors, pregnant women, children, and allogeneic HCT recipients following RSV vaccination.

Figure S2 spectrum.00417-26-s0002.pdf Comparison of anti-RSV-IgG and -IgA antibody activity in allogeneic hematopoietic cell transplant recipients by two commercial ELISAs.

Figure S3 spectrum.00417-26-s0003.pdf Flow chart illustrating concordance in anti-RSV IgA detection by Euroimmun‒ and Virotech‒ELISAs across four study cohorts: healthy blood donors, pregnant women, children, and allogeneic HCT recipients following RSV vaccination.

Figure S4 spectrum.00417-26-s0004.tif Correlation between anti-RSV prefusion F-specific IgG titers and age in children.

Figure S5 spectrum.00417-26-s0005.tif Correlation between anti-RSV IgG antibody activity and IgA concentration measured by Euroimmun‒ELISA.

Figure S6 spectrum.00417-26-s0006.tif Correlation between anti-RSV IgG antibody activity and IgA concentration measured by Virotech‒ELISA.

Table S1 spectrum.00417-26-s0007.docx RSV vaccines and monoclonal antibodies approved or available in Switzerland during cohort enrollment.

Table S2 spectrum.00417-26-s0008.docx Technical characteristics of the anti-RSV immunoassays used in this study.

ASM does not own the copyrights to Supplemental Material that may be linked to, or accessed through, an article. The authors have granted ASM a non-exclusive, world-wide license to publish the Supplemental Material files. Please contact the corresponding author directly for reuse.
