# Supplementary material for: Diagnostic performance and interchangeability of two commercial anti-RSV−IgG and IgA ELISAs across clinically relevant populations
Source: Microbiol Spectr. 2026 Jun 26;14(8):e00417-26. doi: 10.1128/spectrum.00417-26 (PMC13435726; doi:10.1128/spectrum.00417-26)
Supplement: Supplementary file 1 [file spectrum.00417-26-s0001.pdf]

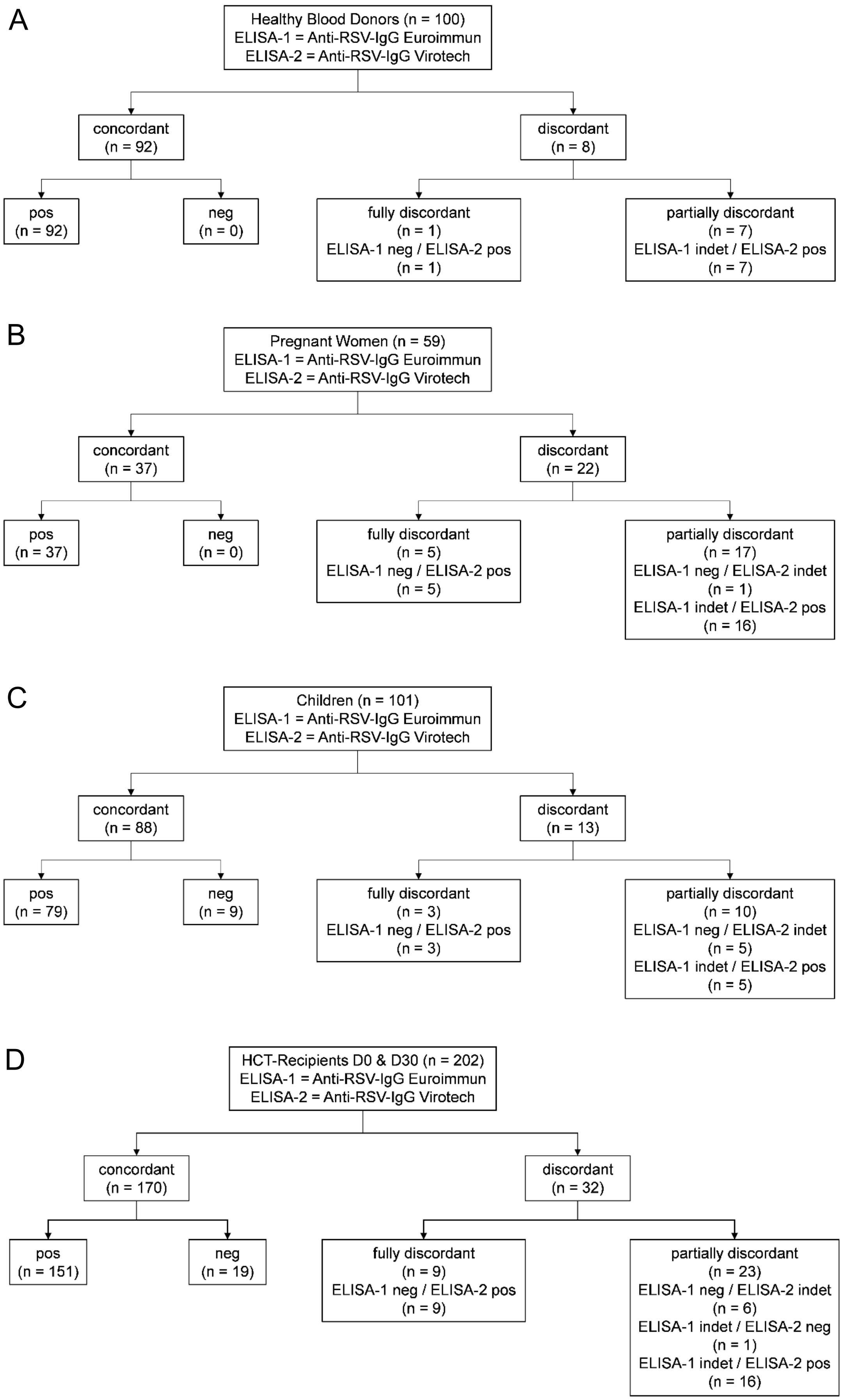

Supplemental Figure 1. Flowchart illustrating concordance in anti-RSV IgG detection by Euroimmun and Virotech ELISAs across four study cohorts, healthy blood donors (A), pregnant women (B), children (C), and allogeneic hematopoietic cell transplant (HCT) recipients following RSV vaccination (D). Abbreviations: pos, positive; neg, negative; indet, indeterminate.
