# Supplementary material for: Diagnostic performance and interchangeability of two commercial anti-RSV−IgG and IgA ELISAs across clinically relevant populations
Source: Microbiol Spectr. 2026 Jun 26;14(8):e00417-26. doi: 10.1128/spectrum.00417-26 (PMC13435726; doi:10.1128/spectrum.00417-26)
Supplement: Supplementary file 2 [file spectrum.00417-26-s0002.pdf]

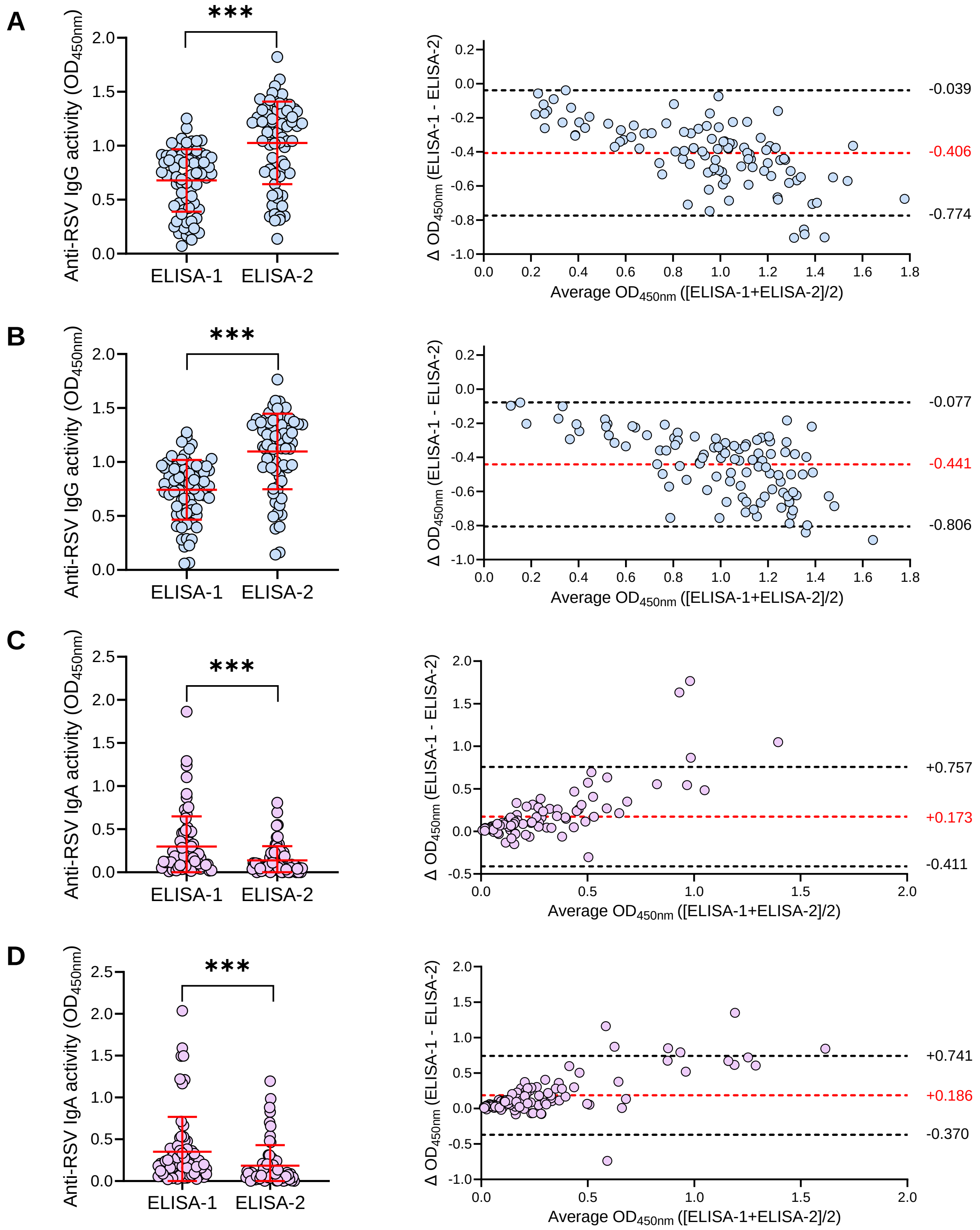

Supplemental Figure 2. Comparison of anti-RSV-IgG and -IgA antibody activity in allogeneic hematopoietic cell transplant recipients by two commercial ELISAs

Optical density (OD_{450nm}) values were measured by Euroimmun Anti-RSV-ELISA (ELISA-1) and Virotech Anti-RSV-ELISA (ELISA-2) in 101 individuals for anti-IgG at baseline (A) and 30 days post-vaccination (B) and for anti-IgA at baseline (C) and 30 days post-vaccination (D). Median optical density values with interquartile ranges are shown, and paired comparisons were performed using the Wilcoxon matched-pairs test (left panels). Bland-Altman analyses are shown, with the dashed red line indicating the mean bias and the dashed black lines indicating ±1.96 standard deviations (right panel).
