# Supplementary material for: Diagnostic performance and interchangeability of two commercial anti-RSV−IgG and IgA ELISAs across clinically relevant populations
Source: Microbiol Spectr. 2026 Jun 26;14(8):e00417-26. doi: 10.1128/spectrum.00417-26 (PMC13435726; doi:10.1128/spectrum.00417-26)
Supplement: Supplementary file 4 [file spectrum.00417-26-s0007.docx]

**Supplemental Table 1. RSV vaccines and monoclonal antibodies approved or available in Switzerland during cohort enrollment**

| Vaccine/Brand | Antigen/monoclonal antibody | Indication | Swiss authorization date |
| --- | --- | --- | --- |
| Arexvy® | RSV pre-fusion F protein (RSVPreF3 antigen) | Active immunisation for prevention of RSV lower respiratory tract disease in adults 60 years and older | 2 May 2024 |
| Abrysvo® | RSV subgroup A and B prefusion F antigens | Active immunisation in adults 60 years and older, and passive protection of infants via maternal vaccination between weeks 32 and 36 of pregnancy | 23 August 2024 |
| Synagis® | Palivizumab | Monthly RSV season prophylaxis for infants at high risk of severe RSV disease | 13 August 1999 |
| Beyfortus® | Nirsevimab | Single-dose RSV prevention for neonates and infants in their first RSV season, and selected toddlers in their second season | Approved in Dec 2023; available in practice from autumn 2024 |
