# Supplementary material for: Diagnostic performance and interchangeability of two commercial anti-RSV−IgG and IgA ELISAs across clinically relevant populations
Source: Microbiol Spectr. 2026 Jun 26;14(8):e00417-26. doi: 10.1128/spectrum.00417-26 (PMC13435726; doi:10.1128/spectrum.00417-26)
Supplement: Supplementary file 5 [file spectrum.00417-26-s0008.docx]

**Supplemental Table 2. Technical characteristics of the anti-RSV immunoassays used in this study**

| **Assay (kit)** | **Detection principle** | **Platform** | **Manufacturer** | **Antigen source** | **Interpretation (cut-off criteria)** | **Reporting unit** |
| --- | --- | --- | --- | --- | --- | --- |
| **Euroimmun anti‑RSV ELISA (IgG)** | Indirect ELISA | Indirect ELISA (microplate with RSV antigens) | EUROIMMUN AG (Lübeck, Germany) | Inactivated RSV antigen (strain “Long”), whole virus lysate from Vero cells | ≥22 RU/mL (positive)  16 to <22 RU/mL (indeterminate)  <16 RU/mL (negative) | Relative Units per mL (RU/mL) |
| **Euroimmun anti‑RSV ELISA (IgA)** | Indirect ELISA | Indirect ELISA (microplate with RSV antigens) | EUROIMMUN AG (Lübeck, Germany) | Inactivated RSV antigen (strain “Long”), whole virus lysate from Vero cells | ≥1.1 RU/mL (positive)  ≥0.8–<1.1 RU/mL (indeterminate)  <0.8 RU/mL (negative) | Relative Units per mL (RU/mL) |
| **Virotech**  **anti‑RSV ELISA (IgG)** | Indirect ELISA | Indirect ELISA (96-well, antigen-coated breakable strips) | Virotech Diagnostics GmbH (Rüsselsheim, Germany) | Inactivated RSV antigen (strain “Long”), whole virus lysate from Vero cells | >11.0 VU (positive)  9.0–11.0 VU (indeterminate)  <9.0 VU (negative) | Virotech Units (VU); 10 VU is the cut-off (internal relative unit calculated from sample optical density vs. cut-off control) |
| **Virotech**  **anti‑RSV ELISA (IgA)** | Indirect ELISA | Indirect ELISA (96-well, antigen-coated breakable strips) | Virotech Diagnostics GmbH (Rüsselsheim, Germany) | Inactivated RSV antigen (strain “Long”), whole virus lysate from Vero cells | >11.0 VU (positive)  9.0–11.0 VU (indeterminate)  <9.0 VU (negative) | Virotech Units (VU); 10 VU is the cut-off (internal relative unit calculated from sample optical density vs. cut-off control) |
| **ACROBiosystems anti-RSV-F0 IgG ELISA** | Indirect ELISA | Indirect ELISA (RSV Pre-Fusion glycoprotein F0-coated microplate; HRP-conjugated secondary antibody) | ACROBiosystems (Newark, DE, USA) | Recombinant RSV pre‑fusion F0 glycoprotein  (RSV subtype A) | Optical density cut-off = 0.1  ≥0.1 positive; <0.1 negative  titer = highest dilution factor yielding a positive reading | Antibody titer |
